# PDE4 Inhibition in Dermatologic Disease: Impacts Beyond Inflammation

**DOI:** 10.1111/ijd.70456

**Published:** 2026-05-17

**Authors:** Keana Khodadad, Nicole E. Chin, Raj Chovatiya

**Affiliations:** ^1^ Rosalind Franklin University of Medicine and Science Chicago Medical School North Chicago Illinois USA; ^2^ Loyola University Stritch School of Medicine Maywood Illinois USA; ^3^ Center for Medical Dermatology + Immunology Research Chicago Illinois USA

**Keywords:** fibrosis, inflammation, phosphodiesterase 4 (PDE4), pigmentation, pruritus

## Abstract

Phosphodiesterase 4 (PDE4) enzymes play a central role in modulating cyclic adenosine monophosphate (cAMP) signaling and regulating diverse cellular responses. Inhibitors of PDE4 have emerged as safe and effective anti‐inflammatory therapies across multiple dermatologic diseases, and growing evidence suggests that PDE4 inhibition exerts broader effects on skin physiology. This review synthesizes mechanistic, preclinical, and clinical evidence describing the multifaceted roles of PDE4 inhibition in the skin. A comprehensive literature review was conducted using PubMed/MEDLINE, Embase, and ClinicalTrials.gov through 2025. Search terms included “PDE4,” “phosphodiesterase 4,” and both approved and investigational therapies, including “apremilast,” “crisaborole,” “roflumilast,” and “orismilast.” Relevant preclinical studies, clinical trials, and mechanistic investigations were included. PDE4 inhibition prevents cAMP degradation and activates protein kinase A (PKA) signaling, leading to broad biologic effects in the skin. Beyond well‐established anti‐inflammatory actions, PDE4 inhibitors appear to influence melanocyte signaling and pigmentation, enhance epidermal barrier protein expression, modulate sensory neuron activity, and promote wound healing by reducing fibrosis. These effects have been demonstrated mechanistically and across multiple dermatologic conditions and may help explain clinical observations. Heterogeneity in study design, limited long‐term data, and variability in PDE4 isoform selectivity across studies may limit generalizability. Further mechanistic and comparative clinical studies are needed to better define therapeutic impact. PDE4 inhibitors represent a unique therapeutic class with pleiotropic effects across multiple disease states, including regulation of pigmentation, barrier integrity, neural signaling, and tissue repair. Their ability to integrate immunologic and non‐immunologic pathways highlights their role in maintaining cutaneous homeostasis and expanding therapeutic potential in dermatology.

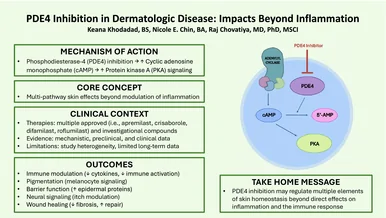

## Graphical abstract

This graphical abstract demonstrates the comprehensive mechanism of PDE4 inhibition in skin physiology. By increasing intracellular cAMP and activating PKA, PDE4 inhibitors exert pleiotropic effects, including immune modulation, pigmentation regulation, barrier enhancement, sensory neuron modulation, and improved wound healing with reduced fibrosis.

## 1 Introduction

Phosphodiesterase 4 (PDE4) enzymes regulate intracellular levels of cyclic adenosine monophosphate (cAMP), a secondary messenger integral to signal transduction pathways [1]. The PDE4 family, composed of multiple isoforms (PDE4 A‐D), hydrolyzes cAMP to 5′‐AMP, controlling downstream activation of cAMP‐dependent protein kinase A (PKA) and exchange proteins directly activated by cAMP (Epac) [2]. In the skin, these pathways influence cytokine production, keratinocyte differentiation, melanocyte activity, and fibroblast proliferation [3, 4].

PDE4 inhibitors have gained prominence as targeted anti‐inflammatory agents in dermatology over the past decade. Oral apremilast is currently approved for plaque psoriasis, psoriatic arthritis, and oral ulcers associated with Behçet's disease, while topical crisaborole and roflumilast are approved for atopic dermatitis, and in the case of roflumilast, psoriasis and seborrheic dermatitis. Beyond these therapies, the clinical landscape of PDE4 inhibitors continues to expand [5, 6, 7].

The anti‐inflammatory role of PDE4 inhibition is well characterized across multiple inflammatory dermatoses. By preventing the breakdown of intracellular cAMP, PDE4 inhibitors enhance downstream signaling through PKA and CREB while suppressing NF‐κB and other proinflammatory transcription pathways [3]. This shift results in reduced production of TNF‐α, IL‐17, IL‐23, IL‐4, IL‐5, IL‐13, and interferon‐γ, cytokines central not only to psoriasis but also to atopic dermatitis, prurigo nodularis, vitiligo, and chronic pruritic conditions. PDE4 inhibition also promotes regulatory immune activity, including increased IL‐10 and regulatory T‐cell associated signaling, contributing to broad immunomodulation. Through these combined effects, PDE4 inhibitors attenuate multiple immune axes (e.g., type 1, 2, and 3 immunity, neuroinflammation), providing therapeutic benefit across a wide range of inflammatory skin diseases [3].

While the anti‐inflammatory actions of PDE4 inhibition are well established, a growing body of research suggests broader roles for PDE4 in skin biology, including effects on pigmentation, epidermal barrier integrity, neuronal signaling, and wound repair [8]. However, these diverse biological functions are often discussed in isolation. A comprehensive synthesis of how PDE4 modulation influences skin structure and function across multiple domains is lacking.

To address this gap, we conducted a narrative review of the literature to closely examine PDE4 signaling and the myriad effects of PDE4 inhibition in dermatology. Through this review, several consistent biological themes emerged, highlighting the multifaceted influence of PDE4 activity on cutaneous physiology beyond inflammation alone.

## 2 Methods

A narrative review of the literature was conducted using PubMed/MEDLINE, Embase, and ClinicalTrials.gov through 2025. Search terms included combinations of “PDE4,” “phosphodiesterase 4,” “apremilast,” “crisaborole,” “roflumilast,” and “orismilast.” Eligible publications included preclinical mechanistic studies, translational research, and clinical trials examining the role of PDE4 signaling or PDE4 inhibition in cutaneous biology or dermatologic disease. Additional references were identified through citation tracking of relevant articles.

Studies were screened for relevance to molecular or cellular effects of PDE4 signaling in skin, biological consequences of PDE4 inhibition, or clinical outcomes related to dermatologic use of PDE4 inhibitors. Findings were qualitatively integrated, and several major recurring mechanistic and biological themes were identified based on patterns that emerged across the literature.

## 3 Mechanism of PDE4 Inhibition

PDE4 enzymes are responsible for hydrolyzing cAMP, thereby inhibiting cAMP‐dependent signaling that regulates the production of inflammatory cytokines [9]. Inhibition of PDE4 leads to intracellular accumulation of cAMP, which activates PKA and Epac1/2. These two cAMP‐dependent pathways have distinct functional roles: PKA primarily drives anti‐inflammatory and immunomodulatory signaling, whereas Epac influences non‐immune cutaneous processes such as melanogenesis, keratinocyte adhesion, and wound healing/remodeling through activation of small GTPases. Because PDE4 isoforms are differentially expressed in keratinocytes, melanocytes, fibroblasts, and immune cells, the relative activation of PKA versus Epac varies across skin compartments, potentially contributing to the diverse clinical effects of PDE4 inhibition [9].

Activated PKA phosphorylates the cAMP response element‐binding protein (CREB), a transcription factor that upregulates anti‐inflammatory mediators such as interleukin‐10 (IL‐10) while suppressing proinflammatory cytokines through inhibition of nuclear factor‐κB (NF‐κB) and nuclear factor of activated T cells (NFAT) [10].

In keratinocytes, melanocytes, and immune cells, this cAMP‐PKA‐CREB signaling axis orchestrates diverse responses. Increased pCREB promotes transcription of genes involved in melanogenesis and cellular differentiation, while decreased NF‐κB activity reduces cytokine‐induced tissue damage. This dual action, consisting of anti‐inflammatory and pro‐regenerative effects, distinguishes PDE4 inhibitors from many conventional immunosuppressants used to treat inflammatory disease [11].

## 4 Anti‐Inflammatory Effects and Clinical Applications of PDE4 Inhibitors

PDE4 inhibitors, including topical agents such as crisaborole, roflumilast, and difamilast, as well as the oral agent apremilast, are established therapies for a range of inflammatory dermatologic conditions. Across clinical trials, these agents consistently demonstrate efficacy in reducing disease severity and pruritus, with generally favorable safety and tolerability profiles [12, 13].

While differences exist in formulation, potency, and PDE4 isoform selectivity [12, 13], their shared mechanism of increasing intracellular cAMP underlies broad immunomodulatory effects [3, 10]. Detailed efficacy and safety outcomes for individual agents have been extensively reviewed in existing literature and are summarized in Table 1.

**TABLE 1:** Current and emerging PDE4 inhibitors in dermatology.

PDE4 inhibitor	Approved or investigational uses	Initial FDA approval date (if applicable)	Reported effects beyond primary anti‐inflammatory clinical data
Apremilast	Psoriatic arthritis [14]	2014 [14]	– Cutaneous hyperpigmentation [15] – Reduces pruritus [16, 17] – Promotes keratinocyte/epithelial barrier function [9] – Enhances fibroblast migration and wound healing [9] – Decreased AD‐related biomarkers for those with moderate‐to‐severe AD [18] – Improvement in ulcerative colititis [19] – Reduced binge‐like alcohol intake and alcohol motivation in mouse models [20]
	Plaque psoriasis [14]	2014 [14]	
	Oral ulcers associated with Behcet disease [14]	2019 [14]	
	Investigational for atopic dermatitis, ulcerative colitis, and alcohol use disorder [18, 19, 21]	N/A	
Cilomilast	Not approved—investigational for COPD [22]	N/A	– Inhibits fibroblast chemotaxis (in vitro, lung injury model) [22]
Crisaborole	Mild to moderate atopic dermatitis (topical ointment) [27]	2016 [27]	– Rapid relief of AD‐associated pruritus [23] – Supports skin barrier function [9] – Promotes skin repigmentation in vitiligo [24] – Reduces neutrophil/macrophage migration to wound sites [25, 26]
Difamilast	Mild to moderate atopic dermatitis (topical ointment) [28]	2026 [28]	– Relief of AD‐associated pruritus [29]
Dovramilast	Not approved—investigational for erythema nodosum leprosum [30]	N/A	– No additional data available
Drotaverine	Not approved in the US—approved as antispasmodic in some other countries [31]	N/A	– No additional data available
E‐6005	Not approved—investigational for AD [32]	N/A	– Decrease in AD‐associated pruritus (murine) [32]
Ensifentrine	COPD [33]	2024	– No additional dermatologic data available – No additional dermatologic data available
GSK‐256066	Not approved—investigational for COPD [34]	N/A	
MK‐0873	Not approved—investigational for rheumatoid arthritis and psoriasis [35]	N/A	– No additional dermatologic data available
Nerandomilast	Idiopathic pulmonary fibrosis [36]	2025	– No additional dermatologic data available
Orismilast	Not approved—investigational for AD, psoriasis and hidradenitis suppurativa [37, 38, 39]	N/A	– Modulates epithelial inflammation markers (human, clinical trials) [37, 38, 39] – Improves PASI/PGA scores in psoriasis (human, clinical trials) [38]
Piclamilast	Not approved—investigational for asthma [40]	N/A	– No additional dermatologic data available
Roflumilast	COPD (oral formulation) [41]	2011 [41]	– Improves skin hypo‐ and hyperpigmentation [42] – Reduces pruritus in AD, psoriasis [43, 44] – Enhances fibroblast‐mediated wound healing [25, 26]
	Plaque Psoriasis (ages > 6 years: topical cream [45], ages ≥ 12: topical foam [46]) Atopic Dermatitis (topical cream) [45] Seborrheic Dermatitis (topical foam) [46]	2023 [45, 46]	
		2024 [45]	
		2023 [46]	
Rolipram	Not approved—has antidepressant properties [47]	N/A	– Induces skin melanization (murine) [48] – Enhances macrophage migration, potentially improving wound repair (in vitro, murine) [48]
Zatolmilast	Not approved—investigational for Fragile X syndrome [49]	N/A	– No additional dermatologic data available

Importantly, beyond their anti‐inflammatory effects, clinical observations across trials, including rapid itch reduction, improvements in skin quality, and normalization of pigmentary changes, suggest that PDE4 inhibition may influence multiple non‐immune pathways [14, 16, 17, 18, 19, 20, 21, 23, 27, 29, 37, 38, 39, 41, 43, 44, 45, 46, 50, 51, 52, 53, 54, 55, 56, 57, 58, 59, 60, 61, 62, 63, 64, 65, 66, 67, 68, 69, 70, 71, 72, 73]. These observations provide clinical context for the mechanistic and translational findings discussed below.

## 5 PDE4 Inhibitor Effects Beyond Inflammation

**FIGURE 1:**
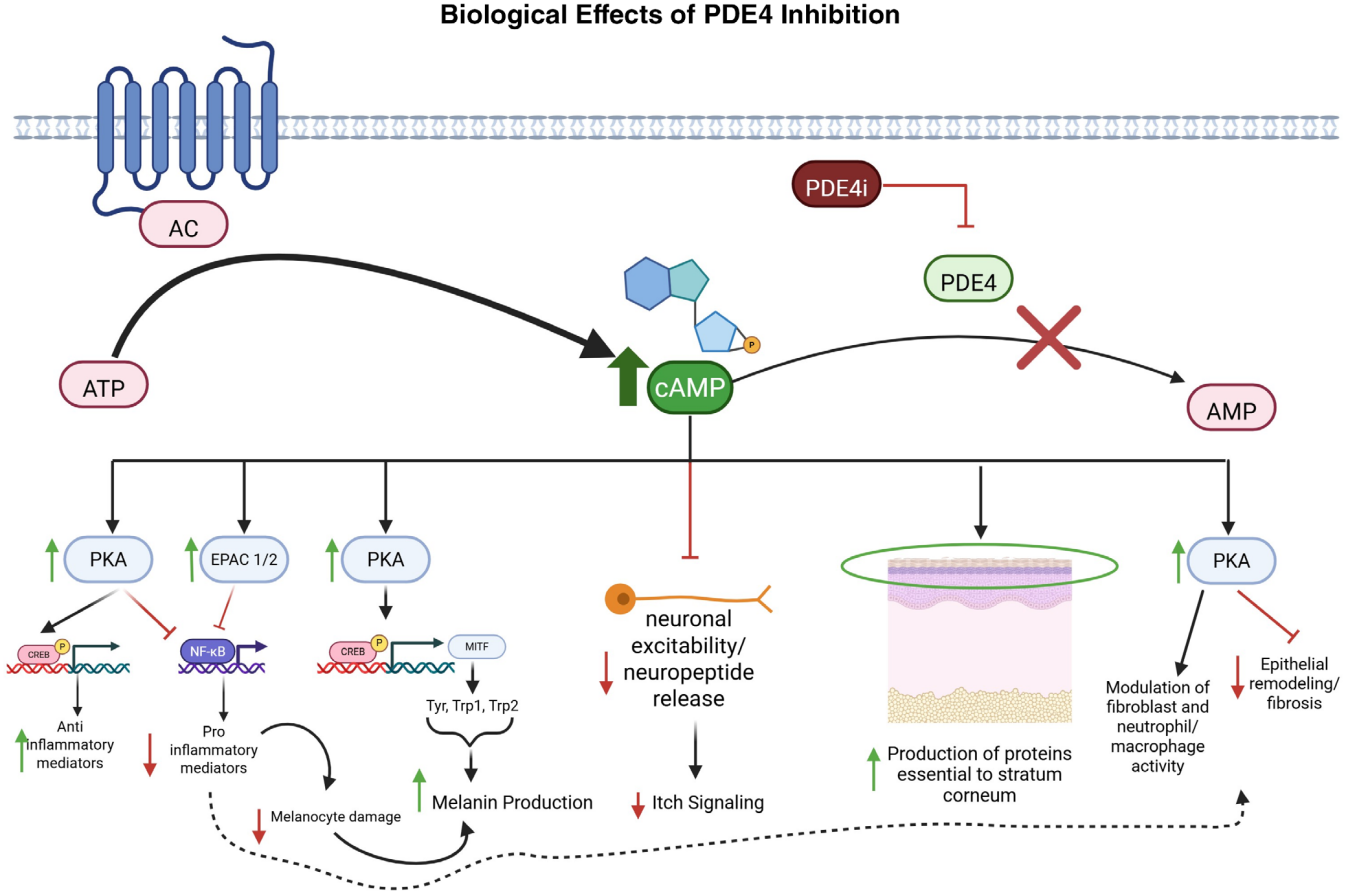
Blocking PDE4 prevents cAMP degradation, resulting in PKA/EPAC activation and suppression of inflammatory transcription pathways. These effects reduce pro‐inflammatory mediator production, decrease neuronal excitability and itch signaling, enhance melanocyte pigment synthesis, and improve epidermal barrier protein expression. Modulation of fibroblast, macrophage, and neutrophil activity contributes to decreased fibrosis and tissue remodeling. Created in BioRender (https://BioRender.com/q32w77d).

Beyond their established role in inflammatory signaling, PDE4 inhibitors influence several other elements of cutaneous biology. When mapped across published studies, these non‐immune effects generally align with distinct functional categories, including pigmentation biology, neuronal activity related to pruritus, epidermal barrier integrity, and tissue remodeling (Figure 1).

To better contextualize findings across the literature, evidence for non‐inflammatory effects of PDE4 inhibition can be categorized into three levels: (1) mechanistic and preclinical data; (2) limited human observational or indirect clinical signals; and (3) established clinical outcomes from controlled trials. Most described effects fall within the first two categories, with relatively limited direct clinical validation from randomized trials at the current time. Additionally, while PDE4 inhibitors share a common mechanism of cAMP modulation, emerging non‐inflammatory effects may not represent uniform class effects. In many cases, available evidence is derived from specific agents, preclinical models, or limited clinical observations, and extrapolation across the entire drug class should be made with caution.

### 5.1 Pigmentation

PDE4 inhibition elevates intracellular cAMP in melanocytes, directly impacting melanogenesis [74]. Preclinical studies demonstrate that cAMP activates PKA, which phosphorylates CREB, leading to transcriptional activation of microphthalmia‐associated transcription factor (MITF), a master regulator of melanocyte differentiation and melanin synthesis [75]. MITF subsequently increases the expression of tyrosinase, tyrosinase‐related protein‐1 (TRP‐1), and TRP‐2, key enzymes responsible for melanin production [48].

In cultured human melanocytes and three‐dimensional skin equivalent models, PDE4 inhibitors enhanced pigment cell proliferation and melanin synthesis, suggesting a direct effect on pigmentation [48]. Another study demonstrated that roflumilast enhances melanogenesis and protects melanocytes from damage secondary to oxidative stress, further supporting a potential protective role in pigmentary homeostasis [75]. Despite these mechanistic findings, clinical outcomes appear to be context dependent. While some reports describe mild hyperpigmentation associated with systemic or topical PDE4 inhibitor use [15], others document cases of repigmentation in disorders such as vitiligo [24]. The net effect likely reflects the balance between direct melanocyte stimulation and the resolution of pigmentary disruption caused by inflammation.

Many pigmentary disorders arise secondarily to inflammation [76]. Postinflammatory pigment alteration (PIPA)—consisting both of post‐inflammatory hyperpigmentation (PIH) and hypopigmentation—can occur following dermatoses such as acne, psoriasis, or atopic dermatitis due to cytokine‐mediated injury to melanocytes and/or disruption of melanin transfer [77]. PDE4 inhibitors suppress key cytokines implicated in these processes, including TNF‐α, IL‐6, IL‐17, and IL‐23 [3], thereby attenuating inflammatory melanocyte damage and reducing PIH. Recent data highlighting the effects of roflumilast foam on normalization of skin pigmentation among patients with seborrheic dermatitis likely reflect these mechanistic findings [42].

Together, these data are supportive of PDE4 inhibitors exerting dual influences on pigmentation: directly through pro‐melanogenic signaling and indirectly through reduction in melanocyte damage.

### 5.2 Neuronal Modulation and Pruritus

The cutaneous nervous system interacts with immune and epithelial cells to regulate sensation and inflammation, and PDE4 enzymes control cAMP levels in each node of this axis. Elevated cAMP dampens neuronal excitability and suppresses neuropeptide release, reducing itch signaling [12, 13]. In atopic dermatitis (AD) and other chronic pruritic dermatoses, cytokines such as IL‐4, IL‐13, and IL‐31 directly sensitize neurons and decrease neuronal cAMP, promoting hyperexcitability [12, 13].

By restoring intracellular cAMP, PDE4 inhibitors counteract cytokine‐driven neuronal sensitization and reduce neuropeptide release [12, 13]. In murine models of AD, a single topical application of the PDE4 inhibitor E6005 significantly reduced scratching behavior and normalized cutaneous nerve activity without affecting mechanical sensitivity; these effects were linked to suppression of basophil IL‐4 production and ERK (extracellular signal‐regulated kinase) phosphorylation, a key player in neuronal activity and communication [32, 78].

These findings are supported by robust clinical trial data, as rapid pruritus reduction is consistently observed across multiple PDE4 inhibitor studies and mechanistic findings parallel observations of rapid itch improvement across PDE4 inhibitor trials: crisaborole demonstrated early pruritus reduction in AD‐301/302 [23, 50]; difamilast studies reported similar findings [79]; and roflumilast produced early improvement in itch in INTEGUMENT‐1/2 and INTEGUMENT‐PED [44, 62]. Early itch reduction is frequently one of the first measurable treatment responses in pruritic skin disorders like AD, underscoring the relevance of neuronal modulation as a therapeutic target.

### 5.3 Skin Barrier and Structural Integrity

The epidermal barrier serves as a critical interface between the body and external environment and is composed primarily of terminally differentiated keratinocytes embedded within a lipid matrix that maintains hydration and protects against mechanical, chemical, and microbial insults [80]. Intracellular cAMP signaling plays a central role in regulating keratinocyte differentiation, lipid synthesis, and tight junction formation, key processes for maintaining stratum corneum integrity and optimal permeability [80, 81].

In inflammatory skin disorders such as AD and psoriasis, cAMP levels are often reduced due to upregulated phosphodiesterase 4 (PDE4) activity, leading to impaired keratinocyte differentiation, increased transepidermal water loss (TEWL), and heightened susceptibility to irritants and microbial invasion [51, 80, 81]. This barrier dysfunction not only contributes to xerosis and inflammation but can also perpetuate immune activation through enhanced penetration of irritants, allergens, and subsequent cytokine release.

Preclinical studies demonstrate that by inhibiting PDE4 and restoring intracellular cAMP, PDE4 inhibitors promote the expression of structural and lipid‐processing proteins critical to epidermal cohesion, such as filaggrin, loricrin, and involucrin [80, 81]. Increased cAMP also enhances the synthesis and organization of ceramides and other key lipids within the stratum corneum, improving hydration and reducing TEWL [80, 81]. Through these mechanisms, PDE4 inhibitors facilitate barrier recovery even in the absence of overt inflammation [51, 80]. The resulting normalization of epidermal differentiation and lipid architecture enhances cutaneous resilience to irritants and pathogens, underscoring the broad therapeutic potential of PDE4 inhibition in disorders characterized by intrinsic or acquired barrier compromise [51, 80, 81].

### 5.4 Wound Healing and Fibrosis

In normal wound healing, a coordinated sequence of inflammation, proliferation, and remodeling restores tissue integrity. cAMP signaling contributes to this process by modulating macrophage activity, fibroblast proliferation, and keratinocyte migration [25, 26]. PDE4 enzymes are expressed in each of these key cell types, where they regulate cytokine production and cellular motility.

Dysregulated PDE4 activity can impair wound healing and promote fibrosis through excessive collagen deposition and aberrant fibroblast‐to‐myofibroblast transition (in addition to sustained inflammatory signaling), mechanisms that underlie hypertrophic scarring, keloid formation, and chronic, non‐healing wounds [25, 26].

Preclinical studies suggest that PDE4 inhibition may enhance tissue repair by increasing intracellular cAMP, which in turn activates protein kinase A (PKA) and Epac pathways. These signaling cascades enhance macrophage migration and phagocytic function, thereby promoting wound debridement and resolution of inflammation [82, 83]. Moreover, PDE4 inhibitors prevent keratinocyte and fibroblast senescence, limit myofibroblast differentiation, and reduce excess extracellular matrix production [83]. Collectively, these effects help facilitate balanced wound remodeling by minimizing fibrosis and improving overall tissue architecture [82, 83]. Given these multifaceted actions, PDE4 inhibition may offer a unique approach for fibrotic and chronic wounds, bridging immunomodulation with structural restoration.

## 6 Knowledge Gaps and Future Directions

Despite a growing body of data describing the broad‐reaching effects of PDE4 inhibition, several gaps remain (Table 2). Most research findings describing pigmentation or barrier outcomes are derived from preclinical models; human evidence is limited to case reports and small series. Future studies should standardize pigment quantification, histologic analyses, and long‐term follow‐up to determine clinical relevance of these preclinical findings. Systematic evaluation of pigmentary changes across larger treatment cohorts through defined endpoints is particularly warranted, as both hypo and hyperpigmentation carry profound psychosocial implications. Similarly, exploration of PDE4 inhibitors in wound healing and fibrosis could yield novel indications beyond traditional inflammatory dermatoses. PDE4 inhibition may also modulate neuronal signaling via cAMP‐dependent pathways, potentially impacting itch, pain, and neuroinflammatory processes. However, clinical and mechanistic data on neuronal outcomes are limited, especially in humans. Future investigations should specifically evaluate neuronal outcomes with oral and topical PDE4 inhibitors to clarify potential therapeutic or adverse effects in the nervous system.

**TABLE 2:** PDE4 Inhibitor Effects in the Skin: Mechanistic and Clinical Evidence.

PDE4 inhibitor effect	Key preclinical/mechanistic data	Key clinical data	Knowledge gaps and future considerations
Anti‐inflammatory/Immuno‐modulation	– ↑ cAMP → activates PKA/CREB, suppresses NF‐κB and NFAT [9, 10] – Reduces TNF‐α, IL‐4, IL‐5, IL‐13, IL‐17, IL‐23, IFN‐γ [3] – Enhances regulatory T cell activity and IL‐10 [3]	Crisaborole – AD‐301/302 (AD) [23] – Stasis dermatitis study [55] – Chronic hand dermatitis retrospective [56] Roflumilast – DERMIS‐1/2 (plaque psoriasis) [43] – ARRECTOR (plaque psoriasis) [60] – STRATUM (seborrheic dermatitis) [61] – INTEGUMENT‐1/2 (AD) [62] – INTEGUMENT‐PED (AD) [44] – INTEGUMENT‐OLE (AD) [63] Difamilast – Phase III trial (AD) [29] Apremilast – ESTEEM 1/2 (plaque psoriasis) [66] – ADVANCE (plaque psoriasis) [68] – SPROUT (plaque psoriasis) [16] – STYLE (scalp psoriasis) [17] – DISCREET (genital psoriasis) [69] – PALACE (psoriatic arthritis) [70, 71] – RELIEF (Behçet's disease) [21] Orismilast – ADESOS (AD) [37] – phase 2b study (plaque psoriasis) [38] – OSIRIS (hidradenitis suppurativa) [39]	– Limited head‐to‐head comparisons, including oral vs. topical – Prospective, long‐term real‐world efficacy, tolerability, safety – Individualized strategies for dosing and/or route of administration
Pigmentation/Melanogenesis	– ↑ cAMP in melanocytes → PKA → CREB → MITF → tyrosinase/TRP1/TRP2 → ↑ melanin [74, 75] – Protects melanocytes from oxidative stress [75] – Observed in cultured melanocytes and 3D skin models [48]	– Case reports of repigmentation in vitiligo (crisaborole) [24] – Roflumilast foam normalized pigmentation in seborrheic dermatitis [42]	– Mainly preclinical data with limited human clinical investigation – Context dependence of hyper‐ vs. hypopigmentation – Lack of standardized pigment quantification in clinical studies
Skin Barrier/Epidermal Integrity	– ↑ cAMP → ↑ filaggrin, loricrin, involucrin [80, 81] – ↑ ceramide synthesis, improved lipid organization [80, 81] – Restores keratinocyte differentiation, reduces TEWL [51, 80, 81]	– Topical PDE4 inhibitors improve barrier function in AD patients (clinically observed reduced xerosis, enhanced hydration) [23, 44, 50, 62, 63]	– Mechanistic contribution of PKA vs. Epac in barrier restoration not fully delineated – Limited pediatric data for barrier endpoints
Wound Healing/Fibrosis	– ↑ cAMP → PKA/Epac → macrophage migration, fibroblast proliferation, keratinocyte migration [25, 26, 82, 83] – Limits myofibroblast differentiation, ECM overproduction [25, 26, 82, 83]	– Primarily preclinical; no robust clinical trial evidence [25, 26, 82, 83]	– Need human trials assessing wound closure, fibrosis, scar prevention – Comparative efficacy of different PDE4 inhibitors unknown
Neuronal/Pruritus Modulation	– ↑ cAMP in neurons → ↓ excitability, ↓ neuropeptide release [12, 13] – Suppresses itch signaling in murine models (E6005, roflumilast analog) [32, 78]	Crisaborole – Rapid itch improvement in AD‐301/302 (AD) [23] Roflumilast – Significant reduction in Worst Itch‐Numeric Rating Scale (WI‐NRS) score in DERMIS 1/2 (plaque psoriasis) [43] – Significant reduction in WI‐NRS and Scalp‐NRS score in ARRECTOR (plaque psoriasis) [60] – ≥ 4‐point WI‐NRS reduction at weeks 1, 2, and 4 in INTEGUMENT 1/2 (AD) [62] – Rapid itch improvement in INTEGUMENT‐PED (AD) [44] Difamilast – Rapid itch improvement in phase III trial (AD) [29] Apremilast – ≥ 4‐point reduction NRS score in ADVANCE (plaque psoriasis) [68] – Decrease in NRS itch score at Week 16 in SPROUT (plaque psoriasis) [16] – Decrease in whole body and scalp NRS in STYLE (scalp psoriasis) [17]	– Few systematic human studies – Oral vs. topical effects on sensory neurons not directly compared
Other Non‐immune Effects (e.g., fibroblasts, keratinocytes)	– PDE4 isoform expression differs in fibroblasts, keratinocytes, melanocytes [9] – May influence proliferation, differentiation, ECM remodeling [9, 25, 80]	– Sparse clinical evidence	– Need PDE4 isoform‐selective studies – Direct clinical data in human non‐inflammatory endpoints lacking

As previously discussed, PDE4 inhibition elevates cAMP and can differentially activate PKA or Epac pathways depending on cellular context and PDE4 isoform expression. Mechanistically, additional work is needed to delineate the relative contributions of PKA versus Epac signaling in non‐immune cutaneous effects. Differences in PDE4 isoform expression between cell types may underlie distinct outcomes in melanocytes, keratinocytes, and fibroblasts. Understanding these nuances could guide the development of isoform‐selective inhibitors with tailored efficacy and safety profiles. Future studies exploring topical and systemic PDE4 modulation in the context of wound repair and scar prevention could further expand their therapeutic relevance.

Among approved agents, apremilast has the most robust clinical data in systemic inflammatory conditions, but less is known about its direct cutaneous effects such as pigmentation or barrier modulation. Topical agents, including crisaborole, difamilast, and roflumilast, have shown efficacy in cutaneous disorders, but clinical data on non‐inflammatory outcomes remain sparse. Mechanistically, PDE4 inhibition elevates cAMP and can differentially activate PKA or Epac pathways depending on cellular context and PDE4 isoform expression. The contributions of these pathways to non‐immune cutaneous effects have not been fully delineated. Differences in isoform expression among melanocytes, keratinocytes, and fibroblasts may explain distinct outcomes, highlighting a need for studies exploring isoform‐selective inhibitors.

## 7 Conclusion

PDE4 inhibitors represent a therapeutic class that integrates immunomodulatory effects with broader influences on cutaneous biology. Through normalization of cAMP signaling, they suppress pro‐inflammatory cascades while also interacting with processes related to melanogenesis, neuronal activity, barrier integrity, and wound repair. The intersection of these pathways underscores the complexity and therapeutic promise of PDE4 inhibition in skin biology. While further mechanistic studies and well‐designed clinical trials are needed to more fully define clinical relevance beyond inflammation, these emerging insights suggest that PDE4 inhibitors may function as broader modulators of cutaneous homeostasis.

## Funding

The authors have nothing to report.

## Conflicts of Interest

R.C. has served as an advisor, consultant, speaker, and/or investigator for AbbVie, Acelyrin, Alumis, Amgen, AnaptysBio, Apogee Therapeutics, Arcutis Biotherapeutics, Argenx, Astria Therapeutics, Avalere Health, Beiersdorf, Boehringer Ingelheim, Bristol Myers Squibb, Cara Therapeutics, Castle Biosciences, Celldex Therapeutics, CLn Skin Care, Dermavant, Eli Lilly and Company, EMD Serono, Formation Bio, Forte Biosciences, Galderma, Genentech, GSK, Incyte, Imagene Bio Inc., Johnson & Johnson, Kenvue, Kowa Pharmaceuticals America, LEO Pharma, L'Oréal, Nektar Therapeutics, Nia Health, Novartis, Opsidio, Organon, Pfizer, RAPT, Regeneron, Sanofi, Sitryx, Takeda, TRex Bio, UCB, Zai Lab. The remaining authors declare no conflicts of interest.

## Data Availability

The data that support the findings of this study are available from the corresponding author upon reasonable request.
